# Looking for Greener Pastures: In Vitro Screening of Phytogenics for the Control of *Sparicotyle chrysophrii* in Gilthead Sea Bream

**DOI:** 10.1111/jfd.70085

**Published:** 2025-11-17

**Authors:** Teresa Pirollo, Ana León, Monica Caffara, Alice Caneschi, Itziar Estensoro, Jinni Gu, Maurizio Scozzoli, Ariadna Sitjà‐Bobadilla, Oswaldo Palenzuela

**Affiliations:** ^1^ Department of Veterinary Medical Sciences University of Bologna Bologna Italy; ^2^ Instituto de Acuicultura Torre de la Sal (IATS, CSIC) Castellón Spain; ^3^ Società Italiana per la Ricerca Sugli Oli Essenziali (SIROE) Roma Italy; ^4^ BioMar AS Trondheim Norway; ^5^ APA‐CT Srl Forlì Italy

**Keywords:** antiparasitic treatments, phytogenics, polyophistocotylean, sustainable aquaculture

## Abstract

The gill parasite *Sparicotyle chrysophrii* poses a significant threat to gilthead sea bream ( *Sparus aurata* ) aquaculture in the Mediterranean, causing considerable mortality and economic losses. As traditional chemotherapeutic treatments, like formalin, face growing regulatory restrictions, there is an urgent need for alternative control strategies. This study screened the in vitro antiparasitic activity of 16 phytogenic active ingredients (AIs), including essential oils (EOs) and commercial feed additives, against adult *S. chrysophrii*. Worms were exposed to a range of concentrations of each compound, and mortality was assessed over 24 h. Lethal dose 50% (LD_50_) values were calculated and categorised by short‐, mid‐, and long‐term efficacy. Several AIs, such as *Cinnamomum zeylanicum* , *Origanum vulgare* , *Thymus vulgaris* , and the commercial formulations Arotec‐G and OA + HE+EO, demonstrated rapid and strong toxicity, with low LD_50_ values observed within 2 h. In addition to lethality, characteristic morphological damage was detected in exposed parasites, indicating a direct and severe parasiticidal effect. These results highlight the high efficacy of various AIs at concentrations significantly lower than those used in standard formalin baths. Overall, the study identifies several promising candidates for the development of alternative treatments against *S. chrysophrii*, providing a solid scientific basis for future in vivo validation and integration into sustainable parasite control programs in marine aquaculture.

## 1 Introduction

In the last decades, Mediterranean aquaculture has experienced exponential growth, primarily driven by advancements in intensive sea cage technologies and industrial hatcheries. However, these farming methods are susceptible to various abiotic and biotic factors that facilitate the spread of parasitic diseases, often resulting in significant mortality and reduced production performance (Fioravanti et al. 2020; Sitjà‐Bobadilla et al. 2006). Among the most widely farmed species in Europe and the Mediterranean is the gilthead sea bream (GSB) ( *Sparus aurata* L.), with an estimated production of 662.3 billion individuals in 2023, yielding 333,000 metric tons at harvest and generating approximately 1.6 billion Euros in first‐sale value (APROMAR 2025). Nowadays, one of the major threats to GSB reared in net pens is the gill‐fluke *Sparicotyle chrysophrii*, responsible for intensity‐dependent mortality and growth retardation (Mladineo et al. 2023).

Like other polyophistocotyleans, this ectoparasite has a direct life cycle and a haematophagous lifestyle (Riera‐Ferrer, Estensoro, et al. 2024). The infection causes severe anaemia, lethargy and gill damage (including epithelial deterioration, necrosis, and inflammation). High parasite loads are associated with signs of hypoxia including increased opercular activity and compromised respiratory efficiency, and often course with severe mortality if untreated (Mladineo et al. 2023; Riera‐Ferrer et al. 2023; Sitjà‐Bobadilla and Alvarez‐Pellitero 2009; Villar‐Torres et al. 2023). Besides the direct mortality, the infection causes reduced body condition factor and it can increase the total feed conversion rate of GSB by more than 0.4, requiring an additional 50,000 tons of feed during the production cycle in Mediterranean production (Rigos et al. 2016). Because temperature influences the development of specific life stages in polyophistocotyleans, global warming is expected to amplify the impact of these infections, particularly given the acceleration of their direct life cycle and the higher pathogenic effect observed at elevated temperatures (Cascarano et al. 2021; Mladineo et al. 2023).

Despite its significant economic and ecological impact, effective control strategies for *S. chrysophrii* in net pen‐based aquaculture settings remain limited. A recent stakeholder survey identified formalin baths (150–300 ppm for 60 min), as the predominant treatment against *S. chrysophrii* in Mediterranean farms. The treatment typically is administered once per production stage (see Oidtmann et al. 2024), rather than at short or regular weekly intervals, and it is usually related to net changes (Oidtmann et al. 2024). However, formalin is increasingly subject to regulatory restrictions, potentially facing EU‐wide prohibition due to concerns related to workplace safety and environmental impact (Oidtmann et al. 2024; Rigos et al. 2023; Wooster et al. 2005). Moreover, the use of formalin baths in net pen systems is both costly and stressful for fish, with variable efficacy, prompting growing interest in alternative control methods (Leal et al. 2018; Tavares‐Dias 2021). Recently, Praziquantel, a widely used anti‐helminthic, has been granted marketing authorization in the EU as a premix for medicated feed for GSB (European Medicines Agency, Committee for Veterinary Medicine 2025). However, its large‐scale effectiveness for the management of sparicotylosis in GSB sea cages still remains unclear, and the use of Praziquantel for the control of other flatworms in aquaculture presents several challenges and limitations (Norbury et al. 2022).

There is increasing global regulation on the use of pharmaceuticals in animal production, supported by international entities such as the Food and Agriculture Organization of the United Nations (FAO), the World Organization for Animal Health (WOAH) and the World Health Organization (WHO). These organisations emphasise the importance of addressing the environmental and public health consequences associated with the overuse of chemicals and medications in disease control (Cabello‐Gómez et al. 2022). In this context, natural compounds represent a promising alternative to reduce the reliance on anthelmintic treatments, offering antiparasitic efficacy with a lower environmental impact (Van Doan et al. 2020; Lieke et al. 2020; Ng et al. 2023). Moreover, their use as in‐feed additives may be subject to fewer regulatory restrictions.

Essential oils (EOs) and various commercial phytogenic formulations – often containing blends of EOs with other active compounds‐ have gained increasing attention in aquaculture for their roles in disease control and growth promotion in farmed aquatic species (Kolygas et al. 2025). These volatile, aromatic substances contain numerous low molecular weight bioactive molecules, typically comprising more than 20 different constituents at varying concentrations. EOs are extracted from various plant parts, including leaves, flowers, and fruits. Although many of the bioactive components are present in trace amounts (< 1%), a few major compounds can account for up to 70% of the oil's total volume and are largely responsible for its biological activity. A growing body of research has shown that plant‐derived EOs and their secondary metabolites exhibit a wide range of biological effects, including antimicrobial, antiviral, antiparasitic, antioxidant, and immunostimulatory properties (Dawood et al. 2021; Van Doan et al. 2020; Harmansa Yilmaz and Yavuzcan Yildiz 2023; Tavares‐Dias 2018), underscoring the importance of analysing EOs and understanding the specific roles of their active constituents.

Studies on the in vitro and in vivo efficacy of natural compounds against *S. chrysophrii* are quite limited. Firmino et al. (2020) reported a significant decrease in *S. chrysophrii* abundance and prevalence using a diet containing microencapsulated essential oils (garlic, carvacrol, and thymol). Additionally, Sitjà‐Bobadilla et al. (2006), assessed the in vitro efficacy of some conventional chemotherapeutics against *S. chrysophrii*, reporting complete adult parasite mortality with hydrogen peroxide (200 ppm) and formalin (300 ppm), whereas praziquantel achieved only 10%–20% mortality at concentrations up to 100 ppm. Among natural compounds, cedrol and curcumin showed the highest toxicity against adult *S. chrysophrii*, although they were significantly less potent than the synthetic reference compound bithionate sodium (Mladineo et al. 2021). More recently, Cabello‐Gómez et al. (2022) reported that the molecules propyl‐propane‐thiosulphinate (PTS) and propyl‐propane‐thiosulfonate (PTSO), derived from onion ( *Allium cepa* ), significantly reduced *S. chrysophrii* larvae survival in vitro, with PTS inducing 90% mortality at 5 μg·mL^−1^.

This research was aimed at identifying and evaluating a wide range of phytogenic AIs and commercial aquaculture in‐feed additives with potential antiparasitic properties, and at assessing their efficacy against adult stages of *S. chrysophrii* in vitro.

## 2 Materials and Methods

### 2.1 Experimental Design and Active Ingredients (AIs)

**FIGURE 1:**
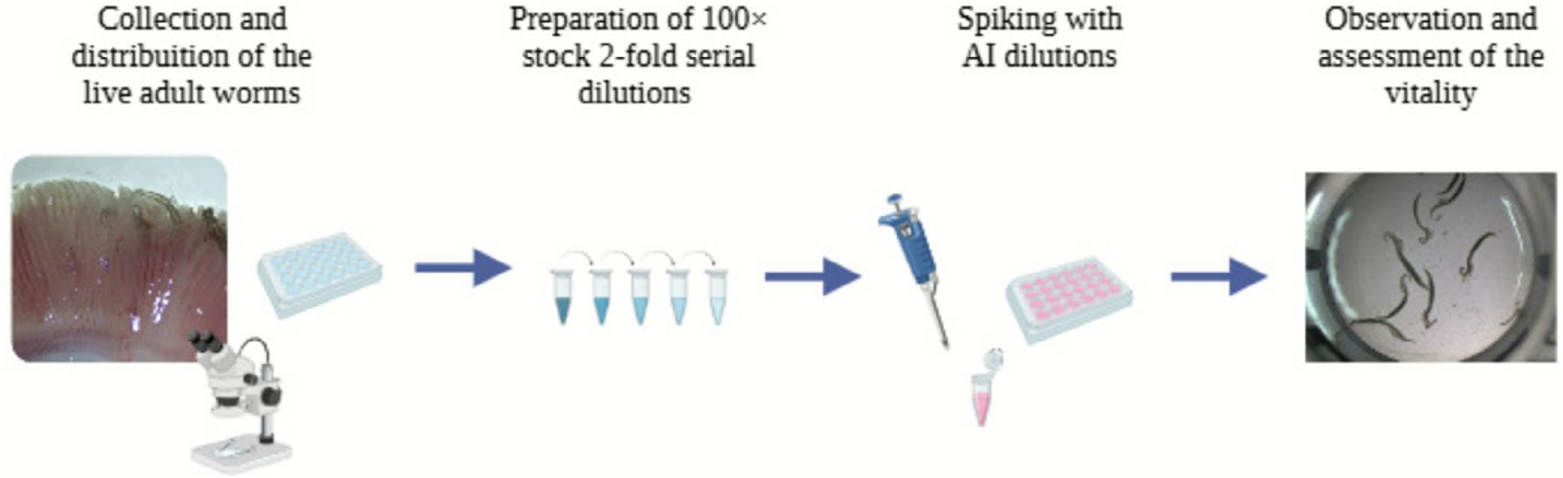
Schematic representation of the in vitro experimental workflow used to assess the effect of active ingredients (AIs) on *Sparicotyle chrysophrii* adult worms.

The in vitro test involved the adult stage of *S. chrysophrii* obtained from experimentally infected fish. Infected stocks were procured through continuous in vivo parasite passages between infected and naïve GSB maintained at the Instituto de Acuicultura Torre de la Sal (IATS, CSIC) facilities, as previously described (Riera‐Ferrer et al. 2023). Briefly, the infection setup consisted of recipient (R) fish tanks containing healthy GSB receiving water from donor (D) fish tanks harbouring infected GSB stocks, within a recirculating aquaculture system (RAS). To enhance the infection process and facilitate monitoring, egg collectors, consisting of a polyester mesh in a supporting plastic frame, were placed in each tank. The collectors trap eggs laid by adult worms and, upon hatching, the free‐swimming parasite larvae (oncomiracidia) reach the fish gills, developing to maturity and subsequently releasing new eggs. This system enables amplification of parasite numbers and non‐lethal monitoring of infection progression. Adult parasites were collected and used for in vitro exposure trials through a multi‐step process (Figure 1). Fish with a high intensity of infection were sampled and sacrificed by overexposure to anaesthetic (MS‐222, 0.1 g L^−1^, Sigma). Gill arches were removed and placed in petri dishes containing sea water; each arch was individually screened under the stereomicroscope, and live adults were carefully detached and temporarily pooled in sterile‐filtered seawater. The live worms were transferred to sterile 24‐well plates, with each well containing 10 worms in 2 mL of sterile‐filtered seawater.

The trial evaluated a range of phytogenic compounds, shortlisted for their reported antiparasitic activity—particularly against flatworms‐ as well as their antimicrobial efficacy and non‐specific immunostimulant or health‐promoting effects in fish. This included both pure essential oils (EOs) and blended commercial formulations marketed as aquaculture feed additives (Table 1). The commercial formulations were provided directly by the producers. All the tested EOs were sourced from GreenVet (Forlì, Italy), and their quality and the amounts of their main active ingredients were routinely assessed using Gas‐Chromatography coupled with flame ionisation detector (GC‐FID). An additional source of *R. officinalis* EO was provided by Delacon Biotechnik GmbH (Engerwitzdorf, Austria).

**TABLE 1:** List of phytogenic active ingredient compounds evaluated in the study, including commercial formulations and essential oils (EOs). Details on the nature of the compound and a summary of applications reported in the literature and focused on fish health are included.

Product name	Characteristics	Active ingredients	Reported effects	References
*Origanum vulgare*	Pure EO	Carvacrol, thymol	Growth and health‐promoter, improves immune responses, in vivo and in vitro efficacy against bacterial infections and monogenean parasites.	Bulfon et al. (2015); Dinardo et al. (2020); García Beltrán et al. (2020); Gonzales et al. (2022); Heluy et al. (2020)
*Melaleuca alternifolia*	Pure EO (Tea tree oil)	Terpinen‐4‐ol (T4O), α‐terpinene	Antimicrobial effect, antiprotozoal and anthelmintic activity, reduces inflammation and improves survival in infected fish.	Baldissera et al. (2017); Carson et al. (2006); Lam et al. (2020); Valladão et al. (2016)
*Citrus lemon*	Pure EO	Limonene, beta pinene	Improves non‐specific immune parameters and growth performance, increases disease resistance against bacterial infections.	Baba et al. (2016); Dawood et al. (2021); García Beltrán et al. (2017); Kesbiç et al. (2022); Ngugi et al. (2017)
Mix gill	Commercial product (blend of EOs)	*Agrimonia eupatoria, Plantago major, Calendula officinalis, C. lemon, Echinacea angustifolia, E* *. globosus* , *Glycyrriza glabra, M. alternifolia, O. vulgare, Solidago virgaurea*	Antibiotic and antifungal properties against microorganisms	Federico et al. (2023)
*Rosmarinus officinalis*	Pure EO	Carvacrol, thymol	Enhances performance in Low‐FM diets, reduces mortality from bacterial infections, improves immune function.	Hernández et al. (2015), Karataş et al. (2020)
*Eucalyptus globulus*	Pure EO	Triterpenic saponins of *Q. saponaria*	Efficacy against bacterial infection, enhances innate and adaptive immune responses.	dos Reis et al. (2025); Nurudeen et al. (2022); Park et al. (2016)
*Menta piperita*	Pure EO	Menthol	In vivo and in vitro efficacy against different species of monogeneans	de Oliveira Hashimoto et al. (2016); da Silva et al. (2024); Malheiros et al. (2016)
*Thymus vulgaris*	Pure EO	Thymol, carvacrol	In vitro efficacy against bacterial infections, enhances immune responses, antiparasitic activity.	Dawood et al. (2021); Gómez‐Mateos Pérez et al. (2017); Tavares‐Dias (2018)
*Cinnamomum zeylanicum*	Pure EO	Cinnamaldehyde, cinnamic acid	Growth‐promoting and antioxidant effects; antiparasitic and antimicrobial activity	Bandeira Junior et al. (2022); Hernando et al. (2025); Hirazawa et al. (2000); Rattanachaikunsopon and Phumkhachorn (2010)
*Lavandula angustifolia*	Pure EO	Linalool, linalyl acetate	Immunostimulant; antiprotozoal and anthelmintic activity	Fazio et al. (2017); Ferreira et al. (2018)
Cineole (Eucaliptol)	Cyclic monoterpene	1,8 cineole	Anthelmintic activity	Zoral et al. (2017)
OA + HE + EO	Mixture of organic acids (OA), inactivated yeast and yeast extracts, herbal extracts (HE) and EO on a mineral carrier (Provided by Adisseo).	Phytobiotics, organic acids and immunostimulants (proprietary mix)	Reduces ectoparasite prevalence in guppy model; enhances skin mucus defenses; supportive against secondary infections.	Nuez‐Ortín and Isern‐Subich (2020)
Garlic and Onion oil	Phytogenic feed additive combining garlic and onion extracts with other bioactive compounds (Provided by DOMCA)	Garlic derivates (allicin)	Effective in vitro and in vivo against *Neobenedenia girellae;* promotes gut microbiota balance.	Van Doan et al. (2020); Ingelbrecht et al. (2020); Naya‐Català et al. (2024); Rimoldi et al. (2023)
Epishield‐Gill	Feed additive with medium‐chain fatty acids, plant extracts (incl. garlic oil), and micronutrients (Provided by Techna)	Plant extracts (including garlic EO), medium‐chain fatty acids and micronutrients (proprietary mix)	Supports epithelial health and enhances wound‐healing processes against parasitic gill challenges.	https://www.groupe‐techna.com/en/feedia/products/epishield‐gill
Arotec‐G	EO‐based feed additive with microencapsulated synthetic garlic, carvacrol, and thymol (Provided by Tecnovit).	Allicin, carvacrol, thymol	Effective against *Sparicotyle chrysophrii*; modulates gut microbiota; improves immune responses; promotes protein biosynthesis in gills.	Firmino et al. (2020); Firmino et al. (2021); Iniesta et al. (2022)

Each concentration of test compound was tested in triplicate wells. For each compound, a broad range of concentrations was tested using a single batch of parasites, as summarised in Table 2. Control wells, run on each plate, contained sterile‐filtered sea water. For non water‐soluble compounds, the control wells contained the same final concentration of diluent present in the highest AI concentration tested (0.1% DMSO).

**TABLE 2:** Concentrations (ppm) of each phytogenic active ingredient tested in vitro with *Sparicotyle chrysophrii*, along with their solubility characteristics. Water‐insoluble compounds were previously dissolved in DMSO and then diluted in sterile sea water to the test concentrations (Con #). The maximum dose of DMSO, present in Con 8 and in the controls, was 0.1%.

Product name	Solubility	Con 8 (ppm)	Con 7 (ppm)	Con 6 (ppm)	Con 5 (ppm)	Con 4 (ppm)	Con 3 (ppm)	Con 2 (ppm)
OA + HE + EO	Oil or EtOH/DMSO	3000	1500	750	375	187.5	93.8	46.9
Garlic and onion oil	Water	1000	500	250	125	62.5	31.3	15.6
Epishield‐Gill	DMSO	4000	2000	1000	500	250	125	62.5
Arotec‐G	DMSO	5000	2500	1250	625	312.5	156.3	78.1
Essential oils	DMSO	500	250	125	62.5	31.3	15.6	7.8

The plates were incubated at 20°C and observed at 1, 2, 4, 6, 20 and 24 h post exposure (p.e.); at each time point, the percentage of live worms in each well was registered; the worms not moving spontaneously were gently poked with a needle and considered dead when no response was observed after 10 s.

### 2.2 Statistical Analyses

Worm survival data were analysed by fitting the survival values for each AI (3 replicate wells per concentration) and time point with a nonlinear fit following an equation [inhibitor] vs normalised response, with variable slope. This model directly uses X values as test concentrations or doses and assumes normalised response Y data between 100% and 0%. Since the evaluated normalised response was worm death, the model Inhibitory Concentration 50 (IC_50_) can be defined as the AI lethal dose 50 or LD_50_. All analyses were performed using GraphPad Prism version 9.4.1 for Windows, San Diego, California USA. Calculated LD_50_ values were categorised into short‐ (< 2 h), mid‐ (4–8 h), and long‐term (> 12 h) exposure groups. This classification provides a simplified overview aimed at identifying AIs with faster toxic effects.

## 3 Results

**FIGURE 2:**
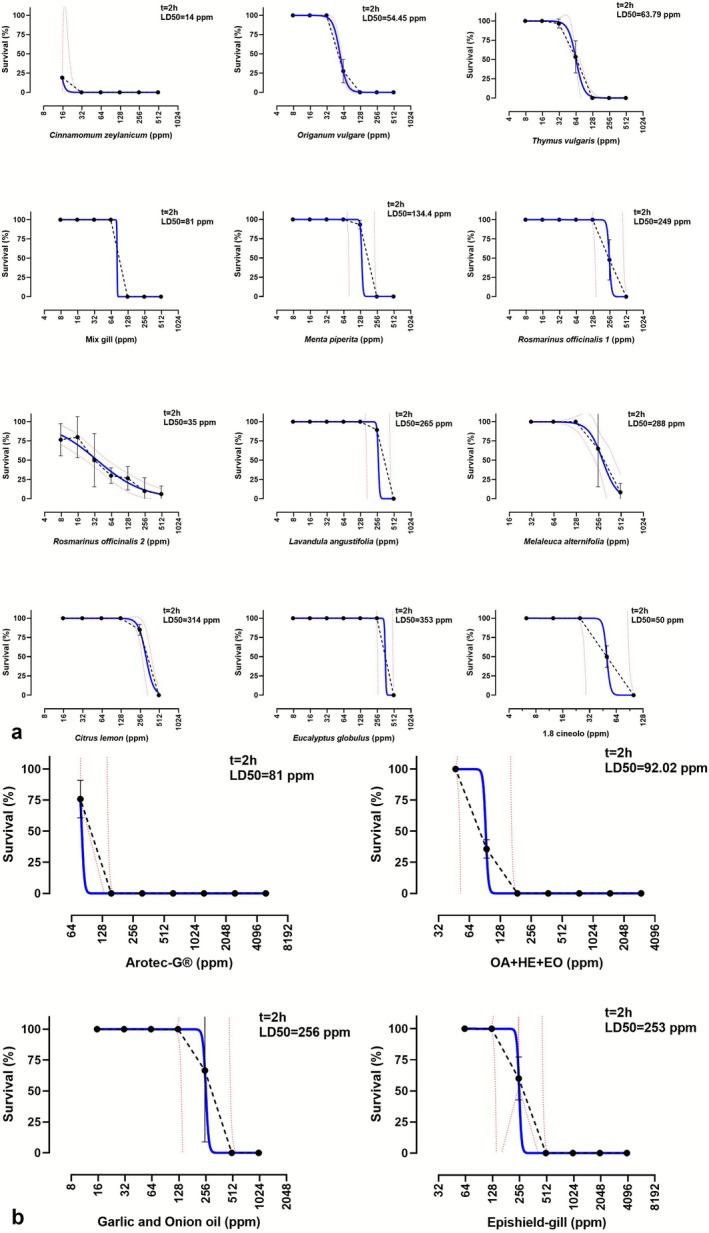
Dose–response analysis of *Sparicotyle chrysophrii* adults survival at 2 h post exposure to natural compounds (a) and commercial products (b) in sea water. Black dots and dotted lines represent the experimental data, with error bars indicate the standard deviation (SD) of replicate wells. Blue lines represent the adjusted nonlinear fit and red dotted lines the corresponding 95% confidence intervals (CI).

Survival of control worms after 24 h was consistently 100%, both with or without 0.1% DMSO. All the tested compounds presented significant toxicity for *S. chrysophrii* in vitro within the tested dose range (Figure 2). However, the AIs differed notably in their estimated LD_50_s and time‐dependent action; in some cases, mortality was observed only at the highest concentration after 24 h (e.g., *Eucalyptus globulus* ), while in others, LD_50_ was reached within the first hour at the lowest tested dilutions (e.g., *Cinnamomum zeylanicum; Origanum vulgaris;* Arotec G and OA + HE+EO). The results of the tests and the calculated LD_50_s for the different AIs are summarised in Table 3. For compounds that caused full mortality at the lowest tested concentrations, accurate estimation of LD_50_ confidence intervals was not possible within the tested dose range. In these cases, the LD_50_ was considered to fall below the lowest concentration tested. Conversely, for compounds with mortality observed only at the highest concentrations and/or longest exposures, dose–response fitting was constrained due to the limited dynamic range of the response curve. LD_50_ values from these fits should be interpreted with caution, as they rely on only a small number of informative data points.

**TABLE 3:** Summary of the in vitro toxicity results of tested phytogenic compounds against *Sparicotyle chrysophrii* adults. The median lethal doses (LD_50_) are categorised into short‐ (< 2 h), mid‐ (4–8 h), and long‐term. *R. officinalis* (1) and (2) refer to the different sources tested. Cell shading intensity correlates with product toxicity (darker green = higher toxicity).

Essential oil	LD_50_ (< 2 h) (ppm)	LD_50_ (4–8 h) (ppm)	LD_50_ (> 12 h) (ppm)
*Cinnamomum zeylanicum*	14	/	/
*Origanum vulgaris*	54	39	8
*Thymus vulgaris*	64	47	8
*Mix gill*	81	72	11
*Menta piperita*	134	145	6
*Rosmarinus officinalis (1)*	249	201	105
*Rosmarinus officinalis (2)*	35	14	6
*Lavandula angustifolia*	265	265	211
*Melaleuca alternifolia*	288	250	179
*Citrus lemon*	314	221	60
*Eucalyptus globulus*	353	353	350
*1,8 cineole*	50	49	27
Arotec‐G	80	80	75
OA + HE+EO	92	81	48
Garlic and Onion oil	250	85–125	68
Epishield‐Gill	250	250	50–100

**FIGURE 3:**
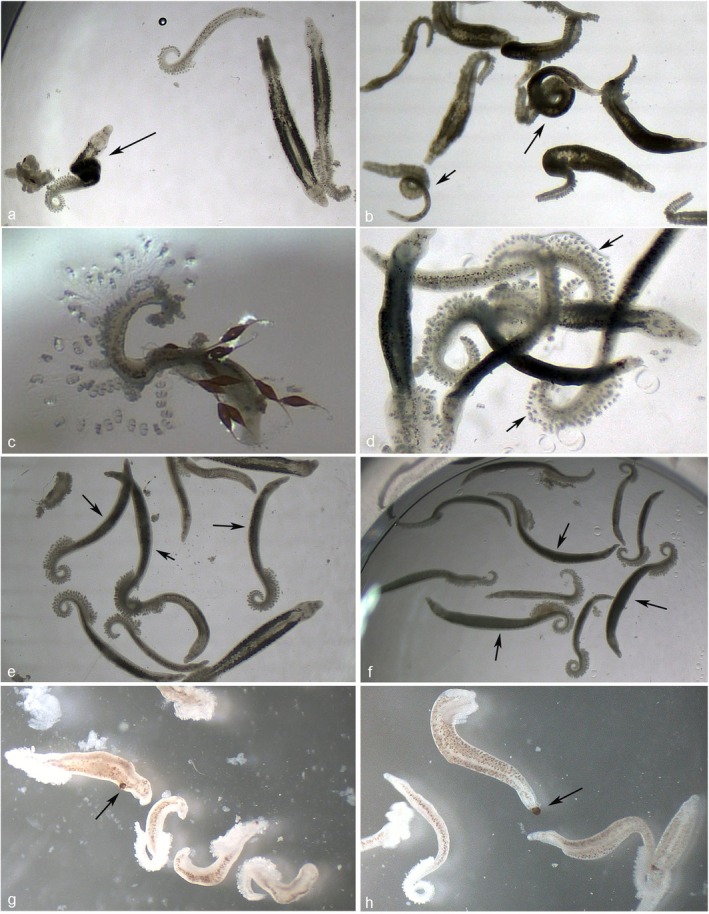
Morphological alterations of *Sparicotyle chrysophrii* adults following treatment with active ingredients (AIs), observed under a stereomicroscope. (a) *Lavandula angustifolia* , 125 ppm, 6 h post‐exposure (p.e.): Adult exhibiting visible torsion of the body (arrow); (b) *Citrus lemon*, 250 ppm, 4 h p.e.: Complete torsion along the entire body (arrows); (c) Mix Gill, 250 ppm, 2 h p.e.: Disruption of the clamps; (d) *Melaleuca alternifolia*, 500 ppm, 1 h p.e.: Dead parasite showing conspicuous, brittle clamps likely to deglove (arrows); (e) *R. officinalis* , 250 ppm, 2 h p.e., and (f) *Thymus vulgaris* , 250 ppm, 1 h p.e.: Darker and swollen appearance of the parasites (arrows); (g) Mix Gill, 62.5 ppm, 4 h p.e., and (h) *Eucalyptus globulus* , 250 ppm, 2 h p.e.: Macroscopic malformation at the anterior end of the parasite (arrow).

In addition to mortality data, parasites exhibited distinct behavioural and morphological alterations following exposure to AIs, particularly at higher concentrations (Figure 3). One of the most frequently observed changes consisted of contraction of the ventral longitudinal muscles, especially conspicuous on the haptoral region, which in some cases progressed to a complete twisting of the parasite's body (Figure 3a,b) ( *R. officinalis* 2, OA + HE+EO, Garlic and Onion oil, *R. officinalis* *1, L* *. angustifolia* , *C. lemon, E* *. globulus* ). In contrast, certain AIs caused relaxation of the worms (e.g., Epishield‐Gill). For certain compounds, the haptor hooklets appeared very conspicuous, brittle, and likely prone to deglove (Figure 3c,d) (Arotec‐G, Mix Gill, *M. alternifolia*). In most treatments, *S. chrysophrii* displayed a swollen, darkened, and opaque appearance (Figure 3e,f) ( *R. officinalis* 2, OA + HE + EO, Garlic and Onion oil, Epishield‐Gill, *C. zeylanicum, O* *. vulgare* , *T* *. vulgaris* , *M. piperita, R. officinalis 1, 1,8‐cineole, C. lemon*). Additionally, macroscopic focal damage, resembling localised swellings, was visible along the parasite's body surface (Figure 3g,h) (*C. zeylanicum, O. vulgare, T. vulgaris, Mix Gill, E. globulus*).

A number of patterns were observed across the tested active ingredients. In particular, Ais characterised by short‐term LD_50_ values below 100 ppm often exhibited steep dose–response curves and a marked increase in efficacy over time. Additionally, behavioural changes in *S. chrysophrii*, such as spasmodic contractions and body shaking, were noted at sublethal concentrations in more than one treatment group, including Mix Gill and *R. officinalis* (1).

Regarding toxicity rankings of individual treatments, *C. zeylanicum* was the most effective EO against *S. chrysophrii*, with a rapid effect at all tested concentrations and the lowest short‐term LD_50_ (14 ppm) among the tested compounds. *T. vulgaris* (LD_50_ 64 ppm) and *O. vulgare* (LD_50_ 54 ppm), as well as one of the batches of *R. officinalis* (1) and 1,8‐cineole, also exhibited strong short‐term effects.

Among commercial formulations, Arotec G and OA + HE + EO were effective at the lowest doses and shorter exposure times (LD_50(2h)_ 80 and 92 ppm respectively). Mix Gill showed a similar short‐term LD_50(2h)_ of 80 ppm.

*M. piperita* showed consistent efficacies in the short and mid‐term (LD_50(2h‐4h)_ 134–145 ppm), but a remarkable increase in lethality in the longer term (6 ppm). *L. angustifolia* (LD_50_ 265 ppm) and *M. alternifolia* (LD_50_ 288 ppm), which required higher doses and longer exposures for significant worm mortality. Other formulations, including Garlic and Onion oil, Epishield Gill, and EOs, like *C. lemon*, and *E. globulus* , showed variable or delayed lethality, with less pronounced short‐term effects. Full data are provided in Table 3.

## 4 Discussion and Conclusion

Validating the antiparasitic potential of EOs and their main constituents represents an essential preliminary step toward their practical application. In vitro assays provide a rapid and cost‐effective approach to confirm antiparasitic activity and to screen a broad range of compounds and concentrations (Tavares‐Dias 2018).

In this study, we evaluated the effects of different phytogenics on the adult stage of *S. chrysophrii*, and we demonstrated remarkable toxicity and low LD_50_, at short exposure times, for several compounds. *C. zeylanicum* EO was the most effective in the short term (LD_50_ < 2 h: 14 ppm). This antiparasitic effect aligns with previous studies, particularly in mammals against *Cryptosporidium parvum* (El Ezz et al. 2011) and *Trichinella spiralis* (Salama et al. 2022). Furthermore, Ling et al. (2015) isolated and tested cinnamaldehyde and cinnamic acid, the two main bioactive compounds of *C. zeylanicum* EO, against the monopistocotylean *Dactylogyrus intermedius*. Both compounds were effective at low concentrations (LD_50_ 48 h: 13.34 and 59.66 ppm respectively) and caused structural damage to the parasite's tegument, as revealed by scanning electron microscopy (SEM) observations. Our results with *S. chrysophrii* confirm *C. zeylanicum* as a promising natural antiparasitic agent effective also against polyophistocotyleans.

*Origanum vulgare* and *T. vulgaris* EOs also exhibited strong activity against *S. chrysophrii* (LD_50_ < 2 h: 54 and 64 ppm respectively). Other studies reported *O. vulgare* extracts resulting lethal for *Trichodina* sp. and *Ichthyobodo* sp. (Mizuno et al. 2018), while *T. vulgaris* EO has been tested against *Trypanosoma* sp., *Echinococcus granulosus*, *Anisakis simplex* and *Ichthyophthirius multifiliis*, showing a wide‐spectrum effectivity across protozoans and helminths (Dardona et al. 2024; Mathiessen et al. 2021). These effects are attributed to thymol and carvacrol, the main bioactive compounds of both EOs. These phenolic monoterpenes are known for their antimicrobial, immunomodulatory, and antiparasitic properties demonstrated in several animal models, including fish (Firmino et al. 2020; García Beltrán et al. 2020; Santoro et al. 2007).

The commercial formulation Arotec‐G, which contains allicin, thymol and carvacrol, showed comparable results (LD_50_ < 2 h: 80 ppm), further supporting the contribution of these bioactive ingredients to the efficacy against *S. chrysophrii*. In a previous in vivo trial, Firmino et al. (2020) reported the efficacy of Arotec‐G in reducing *S. chrysophrii* burden in GSB. This efficacy was linked to the modulation of gill mucosal immunity, including neutrophil‐mediated responses, antioxidant and anti‐inflammatory gene expression, and changes in mucin composition. Indeed, the importance of gill mucosal immunity in the pathogenesis of sparicotylosis has been characterised in recent experimental infection trials (Riera‐Ferrer, del Pozo, et al. 2024, Riera‐Ferrer, Piazzon, et al. 2025; Toxqui‐Rodríguez et al. 2024). The results of the current study demonstrate that different compounds containing these AIs present a remarkable direct parasiticidal effect against *S. chrysophrii*. However, the specific contribution of each isolated AI, their possible synergistic effects, and their mechanisms of action, remain unclear and deserve further investigation.

The results obtained with *Rosmarinus officinalis* (Syn: *Salvia rosmarinus*) are remarkable because important differences in efficacy were found with two sources of this EO. Extracts of *R. officinalis* have previously been found effective against the monopistocotyleans *Dactylogyrus minutus* and *Neobenedenia girellae*, and the polyopisthocotylean *Zeuxapta seriolae* (Ingelbrecht et al. 2020; Zoral et al. 2017). The primary bioactive compound, 1,8‐cineole, is known for its anthelmintic and insecticidal properties, although its proportions in the extracts depend on extraction methods, seasonal variations, and storage conditions. These variables can collectively affect the final composition and purity of the extracted bioactive compounds (Ingelbrecht et al. 2020; Zoral et al. 2017). The short‐term LD_50_ for *S. chrysophrii* determined in our study ranged from 35 ppm to 249 ppm (depending on the EO batch), and the value for pure 1,8‐cineole was 50 ppm. While the quantitative chemical analysis of the most effective batch was not available, the other source contained 34% 1, 8‐cineole and 19% camphor. These results support the effectivity of some *R. officinalis* EOs and particularly of 1,8‐cineole against polyopisthocotyleans, and further highlight the importance of extraction methods, and concentration of bioactive compounds in the treatment outcomes.

The synergistic effect of bioactive compounds found in different EOs has been widely reported (Lebrazi et al. 2025; Soulaimani 2025). This is the rationale behind multiple proprietary commercial blends nowadays marketed as feed additives for animal production. Mix Gill, tested in this study, contains *M. alternifolia* , *C. limon* , and *O. vulgare* EOs, all of them with reported antimicrobial, immunostimulatory, and antiparasitic effects (Alagawany et al. 2020; Harmansa Yilmaz and Yavuzcan Yildiz 2023; Lam et al. 2020; Valladão et al. 2016). While it was not the most effective treatment against *S. chrysophrii* on a short‐term direct in vitro exposure, its LD_50_ ranked among the lowest for commercial products. Other formulations composed of essential oil blends, such as Garlic and Onion oil and Epishield‐Gill, also demonstrated antiparasitic activity, although with higher short‐term LD_50_s. These results are consistent with previous reports (Ingelbrecht et al. 2020) and suggest that, while their direct parasiticidal effect against *S. chrysophrii* appears limited, their action in longer exposures or in‐feed applications requires additional investigation.

Among the less effective products tested in this study were *Mentha piperita*, *L. angustifolia* , *M. alternifolia* , *C. limon* , and *E. globulus* . These EOs exhibited short‐term LD_50_ values ranging between 250 and 350 ppm, although in some cases, such as *Menta piperita* EO, the LD_50_ values were markedly lower with longer‐term exposure. Previous studies have reported antimicrobial, immunostimulant, or antiparasitic properties for these compounds (Carson et al. 2006; Ferreira et al. 2018; Lam et al. 2020; Mohammed et al. 2025; Valladão et al. 2016; Park et al. 2016), and some have shown full efficacy against other pathogens at higher concentrations. These findings suggest that their potential efficacy under longer exposures or in in‐feed applications cannot be ruled out. However, they also highlight that phytogenic active ingredients differ in their mechanisms of actions and in their efficacy against different pathogens.

Direct comparisons between products are challenging due to differences in chemical composition and the range of doses tested. Given the lack of prior efficacy data, it was necessary to include broad concentration ranges, including manufacturer‐recommended levels for products marketed for in‐feed applications. It is also important to note that worm mortality after direct exposure to phytogenics was the only objective parameter to assess efficacy in this study. Sublethal effects were observed for several products, some of which would likely induce severe physiological dysfunctions and promote detachment of the worms from fish at concentrations much lower than their calculated LD_50_.

In the Mediterranean, bath treatments are frequently used to manage gill and skin parasites in caged fish. These treatments require weather‐dependent planning, complex logistics and handling, and the use of large volumes of concentrated formaldehyde. Bath treatments remain among the most costly, labor‐intensive, and environmentally problematic practices in marine aquaculture (Rigos et al. 2023). Given the major impact of *S. chrysophrii* infections in GSB production, alternatives to current practices are urgently needed (Mladineo et al. 2023; Oidtmann et al. 2024; Rigos et al. 2023).

Despite its limitations, this study provides a comprehensive in vitro framework for approaching new, natural alternatives for polyopisthocotylean management. By screening a wide range of popular EOs and phytogenics with alleged antiparasitic effects and determining their actual toxicity for *S. chrysophrii* via direct in vitro exposure, several promising candidates were identified. Notably, some compounds were effective at concentrations much lower than those typically used in formalin baths, highlighting their potential. However, despite these advantages in lower required dosages, the use of essential oils as bath treatments in sea cages will still face the same logistical challenges associated with the mode of delivery, as well as potential cost constraints. The use of synthetic analogues of the most effective ingredients and/or modifications in bath treatment systems (e.g., application via well boats) could result in cost‐effective and sustainable therapeutic tools for polyopisthocotylean management.

Although EOs are often regarded as “natural” and therefore intrinsically safe, toxicological studies have shown that their effective therapeutic concentrations can approach, or even overlap with, lethal levels for some fish species. Acute, sub‐chronic, and chronic exposure tests have demonstrated that high EO concentrations may cause adverse effects, including oxidative stress, gill lesions, and mortality, depending on species, body size, exposure duration, administration route, and temperature (Malheiros et al. 2016; Sumana et al. 2025; Tavares‐Dias 2018). In addition to these safety concerns, another possible limitation relates to the variability and lack of reproducibility in EO‐based products, as observed with the two different *R. officinalis* sources. This chemical variability complicates standardisation and often leads to inconsistent results across different studies (Ng et al. 2023).

Further studies are needed not only to elucidate the mechanisms of action of the most effective ingredients and to establish toxicity thresholds for GSB upon bath exposures, but also to standardise extraction and characterisation methods to ensure consistent chemical composition and reproducible efficacy across EO batches.

Compared to bath treatments, oral delivery of antiparasitic therapies offers clear advantages. However, the development and implementation of in‐feed antiparasitic treatments for *S. chrysophrii* have faced challenges and regulatory restrictions, resulting in a historical therapeutic void in the EU, repeatedly pointed out by researchers and producers. In this context, a 50% premix of PZQ has recently been approved in the EU for the treatment of GSB sparicotylosis. Nonetheless, use of PZQ for flatworm control in sea cages may present challenges and limitations that could limit its large‐scale effectiveness, mainly related to palatability issues and low or unequal food intake (reviewed in Norbury et al. 2022). In addition, while widespread resistance to PZQ has not yet been observed in polyophistocotyleans, reduced susceptibility has been reported in other flatworm taxa, such as *Eubothrium* spp. infecting Atlantic salmon in Norway (Geitung et al. 2021). Finally, PZQ released into the aquatic environment may pose ecological risks, including potential effects on non‐target organisms and sediment accumulation (Ido et al. 2019; Norbury et al. 2022). Although the compound appears to degrade in marine environments, its long‐term ecological impact remains unclear, and systematic studies on persistence and effects in sediments and non‐target species are still lacking (Lunestad et al. 2015; Thomas et al. 2016).

Some of the compounds tested in this work are formulated as feed supplements and, given their toxicity to *S. chrysophrii*, may represent viable alternatives that align with current EU regulatory restrictions on conventional anthelmintic drugs. In this context, understanding the pharmacokinetics of these compounds is critical for determining safe and effective dosages, and further in vitro and in vivo research will be essential to confirm the promising laboratory results, characterise metabolic profiles, assess toxicity and environmental safety, and optimise delivery systems, including in‐feed or controlled‐bath applications, to translate efficacy into realistic field use.

In conclusion, this study provides new data on the in vitro toxicity of 16 plant‐based essential oils and commercial phytogenic formulations against adult *S. chrysophrii*. The LD_50_ values determined across multiple time points highlight several promising candidates for polyopisthocotylean control in aquaculture. Further in vitro and in vivo studies are required to refine dosing protocols, evaluate safety and efficacy, explore potential synergistic effects, and elucidate the underlying mechanisms of action of these bioactive compounds. Integrating these natural products into comprehensive parasite management programs could contribute significantly to developing more sustainable strategies for polyopisthocotylean control in marine aquaculture.

## Author Contributions

**Teresa Pirollo:** conceptualization, data curation, formal analysis, methodology, writing – review and editing; **Ana León:** conceptualization, data curation, methodology, writing – review and editing; **Monica Caffara:** methodology, writing – review and editing; **Alice Caneschi:** resources, draft review and editing; **Itziar Estensoro:** resources, conceptualization, supervision, methodology, writing – review and editing; **Jinni Gu:** resources, draft review and editing; **Maurizio Scozzoli:** resources, draft review and editing; **Ariadna Sitjà‐Bobadilla:** resources, conceptualization, supervision, methodology, writing – review and editing; **Oswaldo Palenzuela:** resources, conceptualization, supervision, data curation, formal analysis, methodology, project administration, writing – review and editing.

## Ethics Statement

Experiments were carried out according to current Spanish (Royal Decree 53/2013) and EU (Directive 2010/63/EU) legislation on experimental animal handling. Procedures were approved by the CSIC Ethics and Animal Welfare Committee (internal code 1499/2023) and by the Regional Authority (Generalitat Valenciana permit number 2023‐VSC‐PEA‐0213).

## Conflicts of Interest

The authors declare no conflicts of interest.

## Data Availability

The data that support the findings of this study are available from the corresponding author upon reasonable request.
